# Endometriosis in Adolescents: A Closer Look at the Pain Characteristics and Atypical Symptoms: A Prospective Cohort Study

**DOI:** 10.3390/jcm14041392

**Published:** 2025-02-19

**Authors:** Maria Federica Viscardi, Ilaria Piacenti, Angela Musella, Laura Cacciamani, Maria Grazia Piccioni, Lucia Manganaro, Ludovico Muzii, Maria Grazia Porpora

**Affiliations:** 1 Department of Maternal and Child Health and Urology, Policlinico Umberto I, Sapienza University of Rome, 00161 Rome, Italy; mariafederica.viscardi@uniroma1.it (M.F.V.); laura.cacciamani@uniroma1.it (L.C.); mariagrazia.piccioni@uniroma1.it (M.G.P.); ludovico.muzii@uniroma1.it (L.M.); 2 Department of Obstetrics and Gynecology, Santa Maria Hospital, 05100 Terni, Italy; ilaria.piacenti85@gmail.com; 3 Department of Maternal and Child Health and Uro-Gynecological Sciences, Policlinico Umberto I, Sapienza University of Rome, 00161 Rome, Italy; an.musella@policlinicoumberto1.it; 4 Department of Radiological, Oncological and Pathological Sciences, Policlinico Umberto I, Sapienza University of Rome, 00161 Rome, Italy; lucia.manganaro@uniroma1.it

**Keywords:** adolescents, chronic pelvic pain, dysmenorrhea, endometriosis, quality of life, hormonal treatment, dienogest

## Abstract

**Background/Objectives**: Endometriosis affects up to 10% of women of reproductive age and about 47% of adolescents with pelvic pain. Symptoms include dysmenorrhea, dyspareunia, and chronic pelvic pain (CPP). Adolescents often present atypical symptoms that can make endometriosis more difficult to diagnose. This study aimed to compare characteristics of pain, atypical symptoms, and the effects of hormonal treatments between adolescents and adults with endometriosis. **Methods**: A total of 238 women with endometriosis were included: 92 aged 12–18 (group A) and 146 over 18 (group B). Data on menarches, cycle length, comorbidities, dysmenorrhea, dyspareunia, CPP, analgesic use, pain characteristics, atypical symptoms, and endometrioma size were recorded. The efficacy, compliance, and side effects of hormonal treatments were also assessed. Quality of life (QoL) was measured using the SF-12 questionnaire at baseline and after six months of therapy. **Results**: Adolescents had earlier menarche (*p* < 0.001), longer menstrual periods (*p* < 0.001), and higher analgesic use (*p* = 0.001) compared to adults. Dysmenorrhea was more frequent (*p* = 0.01), lasted longer (*p* < 0.001), and was associated with higher pain scores (*p* < 0.001) in adolescents. CPP was more common in adolescents (*p* < 0.001), often described as “confined” (*p* = 0.04) and “oppressive” (*p* = 0.038), while adults reported it as “widespread” (*p* = 0.007). Headaches (*p* < 0.001) and nausea (*p* = 0.001) were also more frequent in adolescents. Both groups showed significant improvement in QoL with hormonal treatment (*p* < 0.001) and reported minimal side effects. **Conclusions**: Adolescents with endometriosis often present with earlier menarche, longer menstrual periods, more severe dysmenorrhea, and atypical symptoms. Hormonal contraceptives and dienogest are effective and safe treatments that improve pain and QoL.

## 1. Introduction

Endometriosis is a chronic, estrogen-dependent, inflammatory disease characterized by the presence of endometrial glands and stroma outside the uterine cavity. It affects up to 10% of women of reproductive age [1], and it is mainly associated with symptoms such as dysmenorrhea, dyspareunia, and chronic pelvic pain (CPP) [2,3]. Infertility occurs in 30–50% of cases [4]. In adolescents with pelvic pain, the prevalence of histologically confirmed endometriosis is about 47% [5]. However, the prevalence is likely higher, with estimates reaching up to 75% in adolescents who experience pain unresponsive to analgesics or hormonal treatments [6,7]. Risk factors for early-onset endometriosis include early menarche, low body mass index (BMI), family history, genetic and environmental factors, neonatal uterine bleeding, and Müllerian anomalies [8,9,10]. Inflammation also plays a key role in the pathogenesis of the disease [11,12]. Vitamin D deficiency seems to be associated with pain symptoms, and vitamin D supplementation may be effective for managing both primary and secondary dysmenorrhea in teenagers [13,14,15,16]. Adolescents with endometriosis often report more intense dysmenorrhea compared to adults [17], which is typically described as suprapubic pain radiating to the legs and lower back [18]. This is frequently accompanied by heavy menstrual bleeding in up to 44% of cases or irregular and abnormal uterine bleeding in 60% of cases [9], potentially due to the presence of adenomyosis [19,20]. In young patients, endometriosis may also debut with atypical symptoms [21] such as genitourinary symptoms, nausea, dyschezia, painful bowel movements, constipation or diarrhea, pain during exercise, depression, anxiety, sleep disturbances during menses, migraines, or severe headache [6,17,22,23]. These symptoms manifest earlier in life among adolescents with endometriosis and can debilitate, leading to school absenteeism, negatively affecting social relationships and significantly impairing their quality of life (QoL) [19,21,24,25,26].

The primary objective of this prospective cohort study was to analyze the symptoms and pain characteristics in adolescents aged 12 to 18 years with endometriosis, compared to adult women diagnosed with endometriosis over the age of 18. The secondary objectives were to improve diagnostic accuracy by identifying specific symptoms, thereby facilitating personalized treatments that may delay the need for surgery, optimize pain management, and enhance QoL. Additionally, the study assessed the efficacy, adherence, and side effects of hormonal treatments in adolescents.

## 2. Materials and Methods

Patients with endometriosis referred to the Endometriosis Outpatient Service of Policlinico Umberto I, Sapienza University of Rome, Italy, between 1 January 2021 and 31 March 2024 were enrolled in this prospective cohort study. The study was approved by the Ethics Committee (approval number 0794/2020, approved on 28 October 2020). Informed consent was obtained from all patients or their parents if the patients were underage. Patients diagnosed with endometriosis between 12 and 18 years were included in group A, while those diagnosed over 18 years old were included in group B. Inclusion criteria were age between 12 and 45 years and a first clinical–instrumental (transvaginal/transrectal ultrasound and/or magnetic resonance imaging, where appropriate) diagnosis of endometriosis received within 6 months of recruitment. Exclusion criteria were as follows: pre-menarche or menopause status, prior adnexectomy (unilateral or bilateral) or surgical treatment for endometriosis, active cancer, pelvic inflammatory disease or any other gynecological infections, pregnancy or breastfeeding, and previous or current medical treatment with hormonal contraceptives or progestogens. Each patient’s medical history was carefully evaluated by the same gynecologist at baseline and after 6 months. The following factors were recorded: age at menarche, length of periods, BMI, comorbidities, presence of adenomyosis or deep infiltrating endometriosis (DIE), and size of ovarian endometriomas. The presence, intensity (measured using a 10-point visual numerical scale (VNS)), duration of dysmenorrhea, premenstrual or intermenstrual pain, CPP and dyspareunia, and the use of non-steroidal analgesics (NSAIDs) were recorded. Particular attention was given to the presence of symptoms, such as spotting, heavy menstrual bleeding, mastodynia, gastrointestinal symptoms, bloating, urinary symptoms, headache, low back pain, and nausea. All patients underwent a gynecological examination or, when possible, a transvaginal ultrasound. Alternatively, a transabdominal or transrectal pelvic ultrasound was performed. In selected cases, patients also underwent pelvic MRI. Hormonal treatment with dienogest (2 mg/die) or combined estrogen–progestin therapy (levonorgestrel 0.1 mg/ethinyl estradiol 0.02 mg) administered in a continuous regimen (COC) was proposed. The choice of therapy was based on the patient’s preferences and need for contraception. Data regarding the type of therapy, route of administration, effectiveness, and potential side effects were evaluated both at the time of recruitment and after 6 months. All patients filled in the SF-12 validated questionnaire at the recruitment and after 6 months of treatment to assess the health-related QoL. The scoring system, calibrated to the American population, considers an average score of 50 as indicative of “good health” [27].

### Data Analysis

The sample size was calculated on the basis of an expected prevalence of endometriosis among adolescents with pelvic pain of 25–38%, with a statistical power of 90% and a significance level of 5%. All the data were collected in an Excel worksheet. Statistical analysis was performed using SPSS (version 25 for iMac; IBM, SPSS Statistics, Bologna, Italy). A preliminary descriptive analysis was performed to obtain average values and standard deviations (SD) regarding patients’ general characteristics (continuous variables) and average size of ovarian endometriomas. Quantitative variables were evaluated using the Chi-square test. Normally distributed continuous variables were evaluated using the Student *t*-test, while the non-normally distributed ones were evaluated using the U-test of Mann–Whitney. Statistical significance was set at a *p*-value < 0.05.

## 3. Results

Two hundred and thirty-eight patients were enrolled in this prospective cohort study. Of these, 92 were assigned to Group A (mean age 15.8 ± 1.7 years, range 12–18 years old) and 146 to Group B (mean age 33.9 ± 4.5 years, range 19–45 years old). The general characteristics of the study population are reported in Table 1.

All patients presented with ovarian endometriomas, with an average size of 36.31 ± 22.5 mm in Group A and 29.21 ± 20.3 mm in Group B (*p* > 0.05). Adenomyosis was identified in 4.3% (n = 4) of patients in Group A and 14.3% (n = 21) of patients in Group B (*p* = 0.04). DIE was present in 9.7% (n = 9) of patients in Group A and 17.8% (n = 26) in Group B (*p* = 0.04).

No statistically significant differences were observed in biometric characteristics, urinary and bowel diseases, or medical history, except for a higher prevalence of autoimmune thyroiditis in Group B (9.58%, *p* = 0.035). The main results of the study are presented in Table 2.

Intermenstrual pain was complained of by 39.1% (n = 36) of the patients in Group A and 26% (n = 38) in Group B (*p* > 0.05). Among these, 94.4% (n = 34) of patients in Group A and 84.2% (n = 32) in Group B described the pain as located mainly in the lateral areas of the lower quadrants of the abdomen (*p* = 0.037). Only 11 patients (11.9%) of group A had sexual intercourse and 3 (27.2%) reported deep dyspareunia with an average VNS score of 7.85 ± 2. In group B, 58 patients (39.7%) had deep dyspareunia with an average VNS score of 5.79 ± 2.

Eighty-four patients of group A (91.3%) and one hundred and twenty-one patients of group B (82.8%) received oral hormonal treatment with monophasic, low-dose, continuous COC or dienogest. After six months, only five patients of group A (5.9%) and eight patients of group B (6.6%) had discontinued the treatment due to side effects including vaginal bleeding, headache, and mood changes (more frequently observed among patients taking dienogest) (*p* > 0.05).

**Figure 1:**
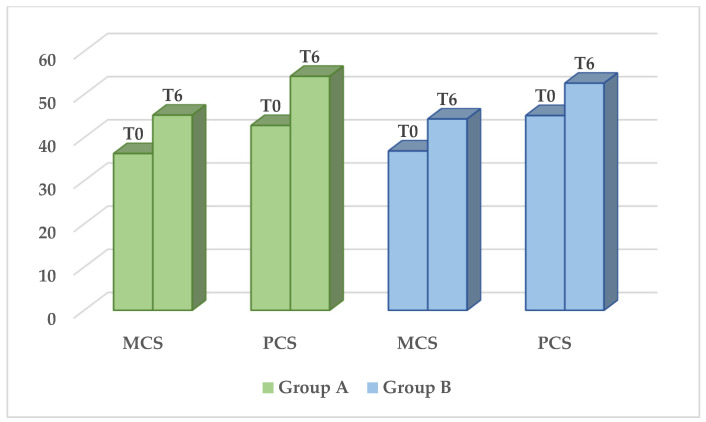
QoL through the means of the SF-12 questionnaire. Significant difference in MCS e PCS (*p* < 0.001) before (T0) and six months after treatment (T6). No statistically significant difference between the two groups. (MCS = mental component score, PCS = physical component score).

The SF-12 questionnaire showed a significant improvement in the QoL in both groups, regardless of the type of treatment. The average mental component score increased from 36.3 ± 11.4 to 45.2 ± 9.9 in group A (*p* < 0.001) and from 36.9 ± 10.1 to 44.3 ± 11.1 in group B (*p* < 0.001). The average physical component score increased from 42.8 ± 10.4 to 54.2 ± 7.9 in group A (*p* < 0.001) and from 45.1 ± 9.7 to 52.6 ± 7.4 in group B (*p* < 0.001) (Figure 1).

## 4. Discussion

Endometriosis is a chronic condition that affects women of reproductive age, often beginning during puberty or in early adolescence. In adolescents, the diagnosis is frequently delayed by up to 11 years, hindering timely identification and treatment and significantly impacting their QoL [20,28]. This delay is largely due to the differences in symptom presentation compared to adults [29]. Dysmenorrhea and pelvic pain can significantly impair school performance, daily activities, relationships, and social engagement [21,24,25,26]. The American College of Obstetricians and Gynecologists recognizes endometriosis as the leading cause of secondary dysmenorrhea that does not respond to treatment with NSAIDs or hormonal suppression in adolescents. They recommend a whole abdominal and pelvic examination (via gynecological visit and transvaginal ultrasound) to rule out other potential conditions, such as obstructive anomalies of the genitourinary tract or bowel diseases [30,31]. However, gynecological examinations or transvaginal pelvic ultrasounds cannot be performed on adolescents who have never had sexual intercourse, and transrectal ultrasound is often unaccepted. MRI may be needed in selected cases to confirm the suspicion of endometriosis [32,33]. Therefore, it is essential to maintain a high index of suspicion and look for signs and symptoms that could facilitate timely diagnosis and treatment, potentially improving pain management and slowing disease progression [9,29]. In this regard, we observed a significant association between confined and oppressive CPP, nausea, and headache in young patients with endometriosis. On the other hand, adults were significantly more often affected by autoimmune thyroiditis, which aligns with the expected increase in autoimmune diseases in older women [34]. Despite evidence suggesting an inverse relationship between endometriosis and BMI [35], we did not find any statistically significant difference between adolescents and adults, possibly due to the small sample size. Similarly, no differences were observed in the size of ovarian endometriomas, in contrast to the findings of Brosens et al., who reported larger ovarian endometriomas in a significant proportion of adolescents with early-onset endometriosis [36]. However, our results align with those of Smorgick et al., who found that most adolescents present with early-stage disease. In addition, we found a low rate of DIE in adolescents according to the literature [36,37]. Nevertheless, our data may underestimate the actual presence of DIE, as several young patients were unable to undergo transvaginal ultrasound, refused transrectal ultrasound, and only a small proportion of them underwent MRI.

A meta-analysis published in 2012 reported that early menarche is associated with a slightly increased risk of endometriosis [8,23]. Consistent with this finding, we observed that adolescents with endometriosis tend to have earlier menarche compared to those diagnosed in adulthood and experience both typical and atypical pelvic pain from their first menstrual period. Additionally, a long menstrual cycle length is a well-established risk factor for endometriosis [38], as it leads to prolonged exposure to menstrual bleeding, increased retrograde flow through the fallopian tubes, and persistence of a pro-inflammatory state within the pelvis [39].

Secondary dysmenorrhea due to endometriosis is often associated with worsening pain, CPP, mid-cycle or acyclic pain, and irregular or heavy menstrual bleeding [4]. This non-cyclical, moderate to severe pain may present as premenstrual and intermenstrual pain, significantly disrupting social activities and daily life. Consistently, we observed a significantly higher prevalence of intermenstrual pelvic pain among adolescents, often accompanied by atypical symptoms. Premenstrual discomfort also presented as headache and nausea in most cases.

Sieberg et al. found that a substantial proportion of young women (one-third) and adult women (64–82%) with CPP had endometriosis, suggesting a link with neuropathic sensitization and neuro-angiogenesis throughout the pelvis [40]. Conversely, Tsonis et al. reported that CPP was more common among younger patients, with an increased risk for disease progression [31]. In our study, both groups experienced CPP, but the intensity was greater in adolescents. Moreover, the characteristics of CPP differed between the groups; young patients commonly reported “localized” and/or “oppressive” pelvic pain, often accompanied by nausea and headaches, whereas adults more frequently described their pain as “widespread.” Similarly, Miller et al. found a higher prevalence of migraine in adolescents with symptomatic endometriosis, particularly those with early menarche. This association appears to be driven by estrogen exposure but, more importantly, by an increased risk of central sensitization [41]. These findings align with previous observations by Di Vasta et al. and Shim et al., who noted that both nausea and headaches were more prevalent among adolescents, significantly impacting their daily lives and social functioning [6,23].

We found no significant difference in the prevalence of dyspareunia between the two groups, although the average VNS score was higher among adolescents. These findings should be interpreted cautiously, as dyspareunia could not be assessed in sexually inactive younger patients.

The observed reduced responsiveness to NSAIDs among adolescents may be attributed to altered leukotriene receptor inhibition, changes in platelet-activating factors, or underlying neuropathic pain mechanisms [42]. Alternatively, it could be due to the use of suboptimal doses of analgesics or to patients’ reluctance to take them before the onset of pain, as recommended by clinical guidelines [30].

Furthermore, Sahin et al. reported a higher prevalence of anxiety and depressive disorders in young women with dysmenorrhea, which may impact pain perception [40,43]. In our study, we did not find any significant difference in mood disorders between adolescents and adults. An impaired QoL was detected by means of the SF-12 questionnaire, but physical and mental component scores improved following medical treatment in both groups.

The hormonal medical treatment with dienogest or COC was discussed with patients from both groups, considering their symptoms, need for contraception, individual preferences, and preferred route of administration. Six months after initiating therapy, patients in both groups reported an improvement in pain, lesion extent, and QoL. Consistent with the existing literature, no significant long-term side effects were observed in young patients using COC or dienogest, further supporting the conclusion that hormonal contraceptives or low-dose progestogens may be a suitable, acceptable, and safe treatment option for symptomatic endometriosis in young patients [29,44,45]. Furthermore, the number of patients who discontinued treatment was very low and not statistically significant. The main side effects reported by these patients were vaginal bleeding, headaches, and mood changes, most observed during the first three months of therapy, especially in patients taking dienogest.

## 5. Conclusions

The results of our study highlight the pain characteristics in adolescents with ovarian endometriosis. A key strength of the study is the observation of “pure symptoms,” unaffected by prior hormonal treatments, pregnancies, or surgeries. However, the small sample size limits the study, making these findings preliminary and requiring validation in a larger cohort.

Adolescents with endometriosis exhibit earlier menarche, longer menstrual periods, more severe dysmenorrhea, and atypical symptoms compared to women diagnosed in adulthood. Their pain is also less responsive to NSAID treatment. They more frequently experience headaches and nausea. CPP in adolescents is often described as “confined” and “oppressive,” with higher VNS scores than those reported by adults. Hormonal contraceptives and dienogest seem to be suitable, safe, and well-accepted treatments for young patients with symptomatic endometriosis, significantly improving both pain and QoL.

The findings of our study provide a basis for further research aimed at the early detection of endometriosis-related symptoms, even in young women. This approach could facilitate earlier diagnosis and tailored treatments, leading to improved clinical outcomes and QoL.

## Figures and Tables

**Table 1:** Study population characteristics.

	Group A	Group B	*p*-Value
Age (average ± SD)	15.85 ± 1.73	33.92 ± 4.5	<0.001
Age at menarche (average ± SD)	11.15 ± 1.36	12.51 ± 1.52	<0.001
Periods length (average ± SD)	6.28 ± 1.64	4.21 ± 0.94	<0.001
BMI (average ± SD)	21.8 ± 3.98	22.32 ± 3.18	ns
Autoimmune thyroiditis n (%)	1 (1.08%)	10 (9.58%)	0.035
Adenomyosis n (%)	4 (4.3%)	21 (14.3%)	0.04
DIE n (%)	9 (9.7%)	26 (17.8%)	0.04
Size of ovarian endometriomas (mm)	36.31 ± 22.5	29.21 ± 20.3	ns

(SD = standard deviation; ns = non significant).

**Table 2:** Prevalence and intensity of symptoms.

	Group A	Group B	*p*-Value
Dysmenorrhea n (%)	90 (97.8%)	120 (82.1%)	0.01
VNS dysmenorrhea (average ± SD)	8.69 ± 1.55	6.98 ± 1.67	<0.001
Pain length (days) (average ± SD)	3 ± 1.49	1.63 ± 0.8	<0.001
Use of NSAIDs n (%)	88 (95.6%)	102 (69.8%)	0.001
VNS after NSAIDs (average ± SD)	4.47 ± 2.2	0.86 ± 1.53	<0.001
Dyspareunia n (%)	3 (27.2%)	58 (39.7%)	0.001
VNS dyspareunia (average ± SD)	7.85 ± 2	5.79 ± 2	0.001
CPP n (%)	58 (63%)	36 (24.6%)	<0.001
VNS CPP (average ± SD)	7.41 ± 2.3	5.61 ± 2.47	0.015
Confined CPP n (%)	42 (72.4%)	16 (44.4%)	0.04
Widespread CPP n (%)	8 (13.7%)	18 (50%)	0.007
Continuous CPP n (%)	14 (24.1%)	6 (16.6%)	ns
Subcontinuous CPP n (%)	4 (6.8%)	0 (0%)	ns
Puntory CPP n (%)	10 (17.2%)	4 (11.1%)	ns
Trafictive CPP n (%)	18 (31%)	6 (16.6%)	ns
Oppressive CPP n (%)	18 (31%)	2 (5.5%)	0.038
Nausea n (%)	40 (43.4%)	9 (13.1%)	0.001
Headache n (%)	48 (52.1%)	12 (8.2%)	<0.001

(SD = standard deviation; ns = non significant).

## Data Availability

The data that support the findings of this study are available from the corresponding author upon reasonable request.

## Abbreviations

The following abbreviations are used in this manuscript:

CPP	chronic pelvic pain
BMI	body mass index
QoL	quality of life
VNS	visual numerical scale
NSAIDs	non-steroidal anti-inflammatory drugs
COC	combined oral contraceptives
ACOG	American College of Obstetricians and Gynecologists
TVUS	transvaginal ultrasound
